# Single- and multiple viral respiratory infections in children: disease and management cannot be related to a specific pathogen

**DOI:** 10.1186/s12879-016-2118-6

**Published:** 2017-01-11

**Authors:** Jérôme O. Wishaupt, Tjeerd van der Ploeg, Ronald de Groot, Florens G. A. Versteegh, Nico G. Hartwig

**Affiliations:** 1 Department of Pediatrics, Reinier de Graaf Hospital, P.O. Box 5011, 2600 GA Delft, The Netherlands; 2 Pieter van Foreest Institute for Education and Research, Medical Centre Alkmaar, Alkmaar, The Netherlands; 3 Laboratory of Pediatric Infectious Diseases, Department of Pediatrics, Radboud University Medical Centre, Nijmegen, The Netherlands; 4 Department of Pediatrics, Groene Hart Ziekenhuis, Gouda, The Netherlands; 5 Department of Pediatrics, Ghent University Hospital, Ghent, Belgium; 6 Department of Pediatrics, Franciscus Gasthuis en Vlietland, Rotterdam, The Netherlands; 7 Department of Pediatric Infectious Diseases and Immunology, ErasmusMC–Sophia, Rotterdam, The Netherlands

**Keywords:** Respiratory tract infections, Child, Co-infection, Respiratory Syncytial Virus, Respiratory viruses

## Abstract

**Background:**

The number of viral pathogens associated with pediatric acute respiratory tract infection (ARI) has grown since the introduction of reverse transcription real-time polymerase chain reaction (RT-PCR) assays. Multiple viruses are detected during a single ARI episode in approximately a quarter of all cases. The clinical relevance of these multiple detections is unclear, as is the role of the individual virus. We therefore investigated the correlation between clinical data and RT-PCR results in children with single- and multiple viral ARI.

**Methods:**

Data from children with ARI were prospectively collected during two winter seasons. RT-PCR testing for 15 viruses was performed in 560 ARI episodes. In the patients with a single-viral etiology, clinical data, laboratory findings, patient management- and outcome data were compared between the different viruses. With this information, we compared data from children of whom RT-PCR data were negative, with children with single- and multiple viral positive results.

**Results:**

The viral detection rate was 457/560 (81.6%) of which 331/560 (59.1%) were single infections and 126/560 (22.5%) were multiple infections. In single viral infections, some statistically significant differences in demographics, clinical findings, disease severity and outcome were found between children with different viral etiologies. However, no clinically recognizable pattern was established to be virus-specific. In a multivariate analysis, the only variables that were correlated with longer hospital stay were the use of oxygen and nebulizer therapy, irrespective of the viral pathogen. Children with RT-PCR positive test results had a significant higher disease severity, fever, length of hospital stay, days of extra oxygen supply, and days of antibiotic treatment than children with a negative RT-PCR test result. For children with single- versus children with multiple positive RT-PCR test results, these differences were not significant.

**Conclusions:**

Disease (severity), management and outcome in pediatric ARI are not associated with a specific virus. Single- and multiple viral ARI do not significantly differ with regard to clinical outcome and patient management. For general pediatrics, RT-PCR assays should be restricted to pathogens for which therapy is available or otherwise may have clinical consequences. Further research with an extended panel of RT-PCR assays and a larger number of inclusions is necessary to further validate our findings.

**Electronic supplementary material:**

The online version of this article (doi:10.1186/s12879-016-2118-6) contains supplementary material, which is available to authorized users.

## Background

Acute respiratory tract infections (ARI) frequently occur in young children. Assessment of disease severity is often difficult and repeated observation over time is recommended [1]. Most ARI’s in young children are of viral origin. Traditionally, clinical guidelines on this subject focus primarily on Respiratory syncytial virus (RSV) and Influenza virus (FLU), as these are considered the most significant viral pathogens [1, 2]. Risk factors for a more severe disease course are best known for RSV [3, 4], although these fail to predict outcome in individual patients. Nowadays, real-time reverse transcription polymerase chain reaction (RT-PCR) assays have been introduced in many hospitals and the number of viruses found in nasal wash specimens (NWS) of children with ARI is growing. The role of many of these viruses in disease severity and clinical course is still unclear, since studies differ with regard to design, age at inclusion, recruitment criteria, the manner of data collection, assay sensitivity and the type of viruses studied [5]. RT-PCR test results are positive in up to 72–95% of symptomatic children and up to 40–68% of asymptomatic children, depending on age, diagnosis and detection method [6]. At the same time, the number of viral co-infections which are detected by RT-PCR has also grown to 43% [6]. Interpretation of these test results is even more challenging. Literature on this subject is growing. Some reports suggest there is no relation between multiple respiratory viral infections and disease severity [7–11], while others report a higher disease severity in children with a multiple respiratory infection [12, 13]. Practical dilemmas about cohorting of patients with different viral pathogens have not yet been solved [14].

In a previous controlled clinical trial, we showed that rapid reporting of RT-PCR test results to the pediatrician did not influence patient care [15]. The aim of the current study was to determine if RT-PCR test results are related to clinical data in children with respiratory symptoms. We investigated clinical symptoms, management and outcome in these children and correlated these findings to the specific virus determined by RT-PCR. We additionally investigated clinical differences between single-, multiple-, and RT-PCR negative ARI.

## Methods

### Study design

This study is part of the EVIDENCE-trial (Evaluation of Viral Diagnostics on Respiratory Infections in Children), a multi-center, controlled clinical trial to evaluate viral RT-PCR diagnostics for ARI in pediatric patients [15]. In summary, the trial was conducted during two consecutive winter seasons (2007–2008 and 2008–2009) in two Dutch teaching hospitals with comparable populations: the Reinier de Graaf Hospital in Delft joined in the second season by the Groene Hart Ziekenhuis in Gouda. The EVIDENCE study-protocol was approved by the regional Medical Ethics Committee (CCMO number NL13839.098.06). In the current study, a selection of the EVIDENCE-dataset is used to analyze the clinical aspects in relation to the viral pathogens.

### Patients

**Fig. 1:**
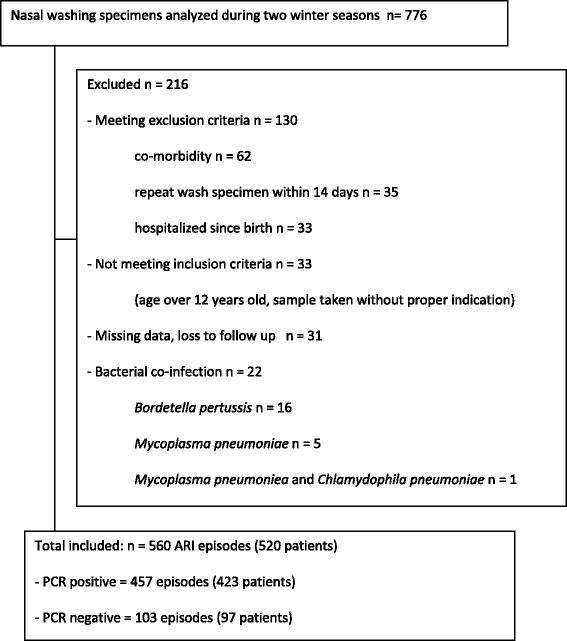
Flowchart of Patient enrollment

Children younger than 12 years old with respiratory symptoms, who visited the emergency department or pediatric outpatient clinic, were included. More than 90% of these children were assessed by the primary physician before referral to the hospital. Informed consent for study participation was sought after the NWS was obtained, because nasal washings are part of standard diagnostic procedures. Indications for hospital admission were made on clinical grounds, e.g. need for extra oxygen, feeding difficulties, apneas as observed by the parents. Children with underlying anatomical airway abnormalities (e.g. bronchopulmonary dysplasia) or other significant underlying disorders (e.g. syndromal disorders, psychomotor retardation, malignancies) were excluded. We also excluded newborns that had been hospitalized since birth. Patients with asthma or suspected asthma were not excluded. Patients could be included multiple times during the two study periods, provided that sampling of NWS was at least 14 days apart to ensure that the children had a new episode of ARI. In addition, patient data were reviewed retrospectively to certify that the sample was taken in a second episode of respiratory symptoms. Patients with positive RT-PCR results for *Chlamydophila pneumoniae*, *Mycoplasma pneumoniae* and *Bordetella pertussis* as single or multiple infection were excluded in order not to trouble comparisons of the virus groups with respect to clinical data. Patients with a positive viral RT-PCR and a clinical confirmed pneumonia were not excluded. Blood cultures or other bacterial cultures were not standard procedures, but were performed on clinical grounds. Patient enrollment criteria are presented in Fig. 1.

### Definitions

ARI was defined as a new episode of respiratory symptoms of the upper and/or lower airways. Upper respiratory tract infection (URTI) was defined as any episode of rhinorrhea, nasal congestion, sore throat, erythematous pharynx, earache or erythematous eardrum. Lower respiratory tract infection (LRTI) was defined as respiratory symptoms with tachypnea and abnormal pulmonary auscultation; rales, crackles, crepitations, wheezing or prolonged expiration. Hypoxia was defined as a pulse oximetric peripheral oxygen saturation of <92% and was not a criterion for LRTI, as it is involved in URTI as well. X-ray confirmation also was not used in the definition, because of a restricted use of ionizing radiation in pediatric practice. Tachypnea was defined by age-dependent cut-off values [16]. Wheeze was defined as high-pitched whistling sound heard coming from the chest on expiration. Apnea was defined as one or more episodes of respiratory pauses regardless of duration observed by caretakers, physicians or nurses. Dyspnea was defined as difficulty of breathing with chest retractions, use of auxiliary respiratory muscles or nose flaring. In single-, dual- and multiple infections, RT-PCR was positive for respectively one, two or more than one virus.

### Data collection

Clinical data were prospectively collected with use of a standardized form by the treating physician. Tables 1, 2 and 3 summarize the data collected. Missing information, laboratory results and, when available, radiology reports were retrieved from the patient’s medical electronic record.

**Table 1:** Demographics, clinical characteristics and disease severity score in single virus infections

	RSV	RV	hMPV	HCoV	FLU	HAdV	HBoV	PIV	*p*-value
*N* = 331 single pathogens	200	30	23	24	21	14	10	9	
Age in months mean (SD)	*5.8 (6.4)*	*5.4(8.5)*	10.2(11.45)	*5.1(6.7)*	**15.1(23.2)**	11.8(11.7)	**18.6(12.4)**	6.8(8.4)	0.001^b^
Median (IQR)	*3.75 (5.56)*	*2.75(5.4)*	4.69(11.84)	*2.67(5.62)*	**2.13(23.53)**	6.4(19.79)	**15.59(24.58)**	5.67(4.87)	
Male, *n(%)*	113(56.5)	18(60.0)	11(47.8)	9(37.5)	11(52.4)	8(57.1)	5(50.0)	4(44.4)	0.638^a,c^
DSS									
mean (SD)	**14.9 (6.9)**	12.6(7.9)	12.2(7.6)	*8.3(6.6)*	*7.9(7.2)*	11.9(8.6)	**19.1(3.4)**	13.9(8.1)	0.000^b^
median (IQR)	**17.0(10.0)**	13.0(15.8)	13.0(16.0)	*7.0,*	*4.0(9.5)*	9.0(18.2)	**19.0(6.5)**	13(16.0)	
DSS ≤6, n(%)	*27(13.5)*	7(23.6)	7(30.4)	**10 (41.7)**	**12(57.1)**	5(35.7)	0(0.0)	2(22.2)	0.000^a,c^
DSS 7–13,n(%)	51(25.5)	10(33.3)	7(30.4)	10(41.7)	5(23.8)	3(21.4)	0(0.0)	3(33.3)	
DSS 14–19, n(%)	78(39.0)	5(16.7)	6(26.1)	3(12.5)	*2(9.5)*	2(14.3)	**7 (70.0)**	2(22.2)	
DSS ≥20, n(%)	44(22.0)	8(26.7)	3(13.0)	1(4.2)	2(9.5)	4(28.6)	3(30.0)	2(22.2)	

*DSS* disease severity score

Significant differences are noted as bold (highest) versus *italic* (lowest) when possible

^a^ Pearson Chi-square tests

^b^ Kruskal Wallis test

^c^ interpret with caution as any cell has a value <5

**Table 2:** Presenting symptoms of single virus infections; parts of the disease severity score

	RSV	RV	hMPV	HCoV	FLU	HAdV	HBoV	PIV	*p*-value
*N* = 331 single pathogens	200	30	23	24	21	14	10	9	
Fever, *n(%)*	108(54.3)	*9(30.0)*	12(52.2)	9(37.5)	**17(81.0)**	7(50.0)	6(60.0)	5(55.6)	0.030^a^
Cough, *n(%)*	**187(94.5)**	24(80.0)	22(95.7)	*13(54.2)*	*14(66.7)*	*10(71.4)*	9(90.0)	8(88.9)	0.000^a^
Rhinorrhea, *n(%)*	190(95.0)	28(93.3)	22(95.7)	22(91.7)	20(95.2)	12(85.7)	10(100.0)	8(88.9)	0.819^a^
Illness > 4 days, *n(%)*	**180(90.0)**	*19(63.3)*	16(69.6)	*13(54.2)*	*10(47.6)*	9(64.3)	8(80.0)	8(100.0)	0.000^a^
Apnea, *n(%)*	7(3.5)	1(3.3)	0(0.0)	1(4.2)	0(0.0)	1(7.1)	1(10.0)	0(0.0)	0.789^a,b^
Wheezing, *n(%)*	**95(47.5)**	13(43.3)	6(26.1)	6(25.0)	*3(14.3)*	6(42.9)	7(70.0)	3(33.3)	0.013^a,b^
Hypoxia, *n(%)*	**105(52.5)**	9(30.0)	9(39.1)	*3(12.5)*	6 (28.6)	5(35.7)	6(60.0)	5(55.6)	0.003^a,b^
Dyspnea, *n(%)*	**136(68.0)**	16(53.3)	15(65.2)	*7(29.2)*	*5(23.8)*	7(50.0)	**8(80.0)**	6(66.7)	0.000^a^
Tachypnea, *n(%)*	123(61.5)	21(70.0)	12(52.2)	12(50.0)	*7(33.3)*	7(50.0)	**10(100.0)**	5(55.6)	0.021^a^

Significant differences are noted as bold (highest) versus *italic* (lowest) when possible

^a^ Pearson Chi-square tests

^b^ interpret with caution as any cell has a value <5

**Table 3:** Outcome, management and laboratory findings in single virus infections

	RSV	RV	hMPV	HCoV	FLU	HAdV	HBoV	PIV	*p*-value
*N* = 331 single pathogens	200	30	23	24	21	14	10	9	
Hospitalization									
admission, *n(%)*	**162(81.0)**	22(73.3)	16(69.6)	*10(41.7)*	16(76.2)	10(71.4)	9(90.0)	7(77.8)	0.004^a^
days, mean (SD)	4.4(2.8)	3.3(3.1)	3.2(1.5)	3.9(3.4)	2.9(2.7)	2.9(2.1)	3.2(1.9)	5.3(2.7)	0.014^b^
median (IQR)	4.0 (4.0)	2.0(3.75)	3.0(2.0)	3.0(3.75)	2.0(1.75)	2.0(1.5)	3.0(1.5)	4.0(5.0)	
Therapy									
AB initiated, *n(%)*	75(37.5)	7(23.3)	7(30.4)	4(16.7)	7(33.3)	1(7.1)	4(40.0)	6(66.7)	0.029^a,c^
AB no of days, mean (SD)	2.4(3.3)	1.4(2.7)	2.4(3.9)	1.5(3.6)	2.0(3.0)	0.5(1.9)	2.8(3.6)	3.7(3.3)	0.091^b^
median (IQR)	0.0(7.0)	0.0(0.8)	0.0(7.0)	0.0(0.0)	0.0(5.0)	0.0(0.0)	0.0(7.0)	3.0(7.0)	
Oxygen initiated,n(%)	106(53)	9(30)	9(39.1)	3(12.5)	6(28.6)	5(35.7)	7(60)	5(55.6)	0.062^a,c^
Oxygen no of days, mean (SD)	2.4(2.7)	1.2(2.2)	1.5(1.7)	1.0(1.8)	0.9(1.5)	1.4(2.1)	1.6(1.3)	2.9(2.9)	0.052^b^
median (IQR)	2.35(4.0)	0.0(2.5)	1.0(3.0)	0.0(2.3)	0.0(1.8)	0.5(2.5)	2.0(2.0)	3.0(5.0)	
Nebulization^d^ initiated n(%)	63(31.5)	6(20)	6(26.1)	0(0)	3(14.3)	4(28.6)	6(60)	3(33.3)	0.086^a,c^
Nebulization^d^, no of days, mean (SD)	1.2(2.0)	0.7(1.5)	0.8(1.3)	0.0(0.0)	0.4(0.9)	1.4(2.1)	2.2(2.2)	1.3(1.6)	0.050^b^
median (IQR)	0.0(2.0)	0.0(1.0)	0.0(1.75)	0.0(0.0)	0.0(0.0)	0.0(2.75)	2.0(3.0)	0.0(3.0)	
Laboratory									
CRP performed, n(%)	111(55.5)	16(33.0)	9(39.1)	14(58.3)	15(71.4)	7(50.0)	4(40.0)	2(22.2)	0.401^a,c^
CRP max level, mean (SD), mg/L	23.1(25.4)	13.8(19.9)	71.0(117.7)	19.0(44.4)	16.6(18.8)	27.3(36.7)	23.7(26.3)	26.0(31.1)	0.440^b^
median (IQR)	14.0(37.0)	5.0(25.5)	14.0(112.0)	0.0(14.5)	9.0(25.0)	7.0(63.0)	21.0(48)	26.0(44.0)	
CRP > 40, n(%), mg/L	27(13.5)	2(6.7)	3(13.0)	2(8.3)	2(9.5)	2(14.3)	2(20.0)	1(11.1)	0.706^a,c^
WBC performed, n(%)	26(13.0)	3(10.0)	1(4.3)	0(0.0)	8(38.1)	1(7.1)	1(10.0)	1(11.1)	0.028^a,c^
WBC max level, mean (SD), 10^9^/L	11.6 (4.5)	9.5 (2.4)	7.6	.	8.1 (2.9)	12.7	13.6 (0.3)	27.0	0.065^b^
median (IQR)	11.1(6.1)	9.4	7.6(0)	.	8.8(5.6)	12.7(0.0)	13.6(0.0)	27.0(0)	

*AB* antibiotics, *CRP* complement reactive protein, *WBC* white blood count

Significant differences are noted as bold (highest) versus *italic* (lowest) when possible

^a^ Pearson Chi-square test

^b^ Kruskal Wallis test

^c^ interpret with caution as any cell has a value <5

^d^ Nebulization with salbutamol and ipratropium bromide

### Disease severity score (DSS)

The DSS used in this study is a modification of the one used by Gern et al. [17, 18] (Additional file 1: Table S1). In the original score, cough and rhinorrhea are subdivided in mild, moderate and severe. We could not make that subjective distinction in our dataset. Hoarseness was also not included in our score. In the original score, the maximum was 31; in our modified score the maximum is 27.

### Respiratory pathogens

All samples were tested for RSV with a rapid bedside test and supplementary RT-PCR assays were performed for 15 viruses and 2 bacteria (*Chlamydophila pneumoniae* and *Mycoplasma pneumoniae*). RT-PCR for *Bordetella pertussis* was performed only on clinical suspicion and retrospectively in all available samples [19]. A description of the RT-PCR method and validation procedure is published elsewhere [15]. Viral subtypes were clustered into virus groups in order to have sufficient patient-numbers in each virus group. RSV-A and RSV-B were clustered. Human Coronavirus (HCoV) 229E, HCoV-NL63 and HCOV-OC43 were clustered. FLU-A and FLU-B were clustered, as well as Parainfluenza virus (PIV) 1, 2, 3 and 4. Other viruses included rhinovirus (RV, not divided in subgroups), Human Metapneumovirus (hMPV), Human Adenovirus (HAdV) and Human Bocavirus (HBoV). We did not study SARS Coronavirus, Human Coronavirus HKU1, enterovirus, Polyomavirus WU and KI.

### Other diagnostic procedures

Other diagnostic tests were only performed on clinical grounds: white blood count, C-reactive protein (Table 3), blood cultures and X-rays (data not shown).

### Statistical analysis

Statistical analysis was performed using IBM SPSS Statistics 21.0 (SPSS inc., IBM Company, Chicago, Illinois). For the comparison of categorical or dichotomous variables with the pathogen groups, we used Pearson Chi-squared tests. For the comparison of continuous variables, we used Kruskal-Wallis- and MannWhitney tests. For all tests, a *p*-value <0.05 was considered significant. Multiple regression analysis was used to analyze the relation between age, DSS, LRTI, antibiotic initiated, number of days with antibiotics, number of days with extra oxygen, number of days with nebulization, the virus groups and the outcome days in hospital. A *p*-value <0.05 was considered as significant.

## Results

### Patient enrollment

During the two study periods, a total of 776 NWS were analyzed. 216 were excluded. In total, 560 viral ARI episodes (520 patients) were analyzed (flowchart, Fig. 1).

### Demographics

The mean age in this study was 7.9 months and 60.5% was male. Single- and multiple infections did differ significantly in age (7.3 versus 9.0 months, *p* < 0.001), sex (54.1% versus 64.3% male, *p* = 0.049) and daycare attendance (30.8% versus 48.0%, *p* = 0.002). Patient reported family history of an atopic constitution was 56.5% and did not significantly differ between the virus groups.

### Viral results

The detection rate of viruses by RT-PCR was 457/560 (81.6%) (Table 4). Single-infections were detected in 331 out of 560 (59.1%) ARI episodes. Multiple infections were detected in 126/560 (22.5%) episodes of which 106/560 (18.9%) were dual infections, 18/560 (3.2%) were triple infections and 2/560 (0.4%) were quadruple infections. A negative RT-PCR was present in 103/560 (18.4%) episodes.

**Table 4:** RT-PCR Results in children with acute respiratory tract infections

RT-PCR results	N	Proportion out of total (*n* = 560 cases)	Detection in single infections	Proportion out of total single infections (*n* = 331)	Detection in multiple infections	Proportion out of total multiple infections (*n* = 126)
Negative	103	18.4%				
Single	331	59.1%				
Dual	106	18.9%				
Triple	18	3.2%				
Quadruple	2	0.4%				
RSV	291	52.0%	200	60.4%	91	72.2%
RV	72	12.9%	30	9.1%	42	33.3%
HCoV	71	12.7%	24	7.3%	47	37.3%
HAdV	45	8.0%	14	4.2%	31	24.6%
hMPV	42	7.5%	23	6.9%	19	15.1%
FLU	33	5.9%	21	6.3%	12	9.5%
PIV	29	5.2%	9	2.7%	20	15.9%
HBoV	22	3.9%	10	3.0%	12	9.5%

*RSV* Respiratory Syncytial Virus, *RV* Rhinovirus, *HCoV* Human Coronavirus, *HAdV* Human Adenovirus, *hMPV* Human metapneuvirus, *FLU* Influenzavirus, *PIV* Parainfluenza virus, *HBoV* Human Bocavirus

RSV was positive in 200/331 single infections (60.4%), 78/106 (73.6%) dual infections and 91/126 (72.2%) multiple infections. RSV was positive in all of the most frequent combinations of dual infections (data not shown).

**Fig. 2:**
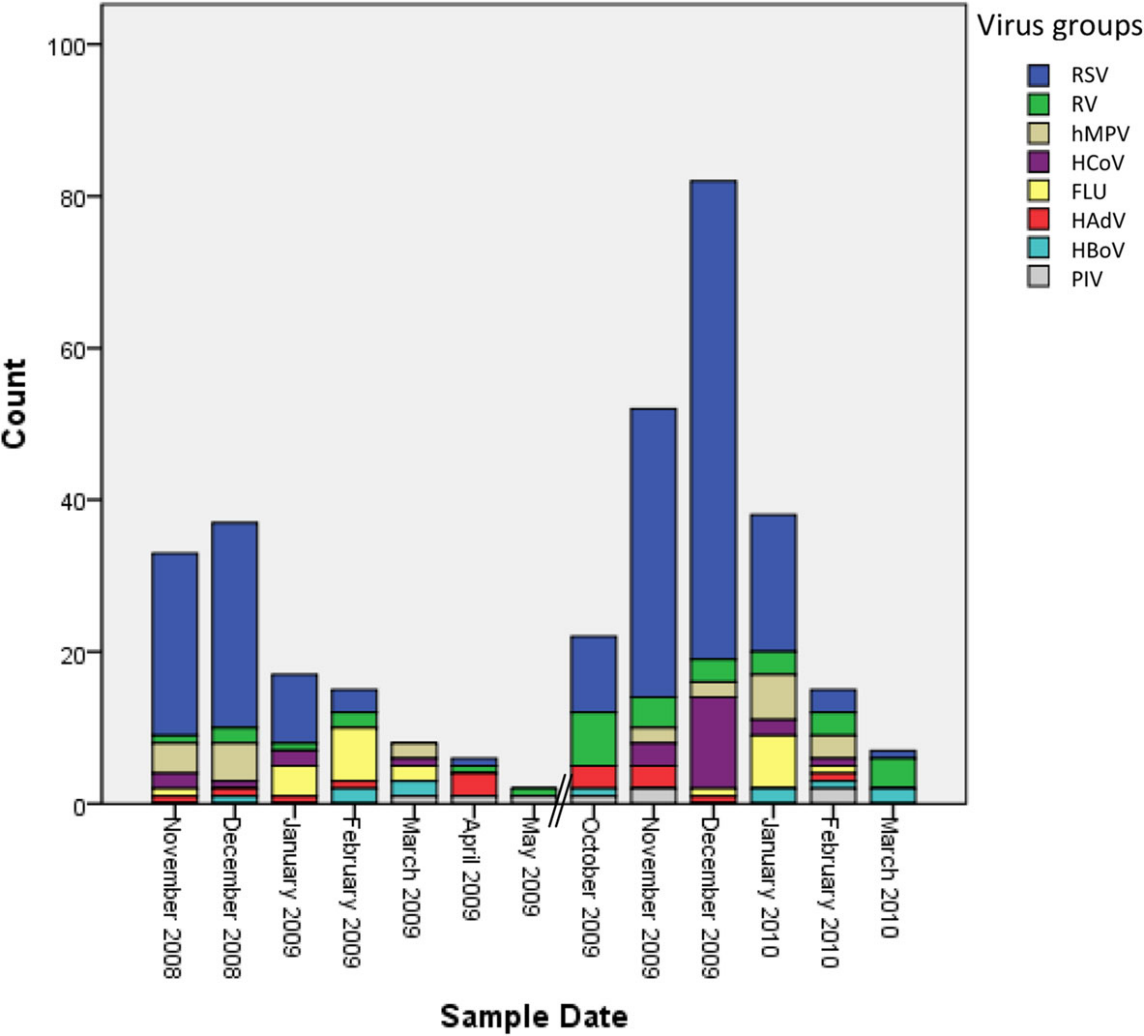
Distribution of virus groups: count per month. Abbreviations: RSV, Respiratory syncytial virus. RV, rhinovirus. hMPV, Human Metapneumovirus. HCoV, Human coronavirus. FLU, Influenza virus. HAdV, Human adenovirus. HBoV, Human bocavirus. PIV, parainfluenza virus

The distribution of the viruses per month is shown in Fig. 2. Peak incidence of FLU in both seasons was in January and February. Other viruses were isolated throughout both winter seasons.

The original study was a randomized controlled clinical trial [15]. A chi-square test showed an equal distribution of the virus groups between intervention- (rapid reporting of PCR-results to the clinician) and the control (late reporting) group (data not shown).

### Clinical symptoms and management of single viral infections

The overall admission rate in single viral infections was 252/331 (76.1%). Extra oxygen supply was administered to 149/252 (59.1%) and nebulizer therapy to 91/252 (36.1%) hospitalized children. Although the p-value indicated a significant difference between the virus groups with regard to the number of times that antibiotics were initiated, it was not possible to explore this difference using the Pearson Chi-square test. There were no significant differences between the virus groups with regard to the mean number of days with antibiotic treatment, extra oxygen supply, nebulizer therapy and the number of children with feeding problems due to respiratory distress, resulting in a need for tube feeding. There were also no significant differences between the virus groups regarding mean or maximum CRP count and mean or maximum white blood count (Table 3).

The multivariate analysis for length of hospital stay (LOS) included age, DSS, LRTI, antibiotic treatment, oxygen therapy, nebulizing therapy and single virus groups. In the univariate analysis, RSV and RV were the virus groups that were correlated with longer hospital stays. In the multivariate analysis, the only variables that were correlated with longer hospital stays were oxygen therapy and nebulizer therapy, irrespective of the viral pathogen. For DSS, there was a significantly adjusted *p*-value, whereas the multivariate regression coefficient was negative (Table 5). In a multivariate sub analysis for oxygen therapy including RSV, RV and FLU, RSV was significantly correlated with longer duration of oxygen therapy (*p* = 0.020). For nebulizer therapy and duration of antibiotic treatment, there was no significant correlation with these viruses (data not shown).

**Table 5:** Multivariate analysis in single viral respiratory tract infection with regard to Length of Hospital Stay

	Univariate		Multivariate	
Variable	Spearman’s ρ	*p*-value^a^	Regression coefficient	Adjusted *p*-value
Age	−0.096	.128	−0.019	0.165
DSS	0.371	<0.01	−0.038	0.052
AB initiated	0.334	<0.01	0.352	0.571
AB no of days	0.336	<0.01	0.026	0.769
FiO2 no of days	0.671	<0.01	0.885	<0.01
Nebulizer	0.120	<0.01	0.293	0.005
RSV		<0.01	0.306	0.226
RV		0.022	0.409	0.324
hMPV		0.446		
HCoV		0.623		
FLU		0.024		
HAdV		0.125		
HboV		0.470		
PIV		0.128		

^a^ For continuous variables, Mann-Whitney *U* tests were used

The characteristics per virus group are presented in Tables 1, 2 and 3 and are highlighted per virus group below.

- *RSV:* RSV was the most frequently detected virus and was found in 200 out of 331 (60.4%) single infections. The mean age of children with RSV was 5.8 months and they were significantly younger than children with FLU or HBoV. RSV positive children were significantly more often hospitalized than HCoV positive children. The DSS for children with RSV and HBoV was significantly higher than for those with HCoV and FLU. The mean DSS for RSV was 14.9, the second highest after HBoV (DSS 19.1). This was significantly higher than for instance FLU (DSS 7.9). Apneas occurred in 7/200 (3.5%) of RSV single infections. One child was RT-PCR positive for RSV as single pathogen, despite a first vaccination with palivizumab. It was a 2 month old boy born after 32 weeks of gestation with a mild disease course (DSS 13, LOS 4). None of the children in the study were treated with the antiviral drug ribavirin.
- *Rhinovirus:* RV was the second most commonly identified single virus infection (30/331, 9.1%). The mean age of children with RV was young; 5.4 months. The DSS was not significantly different compared to those of other viruses.
- *Human metapneumovirus:* For hMPV, there were no significant differences compared to the other viruses with regard to age, DSS and admission rate.
- *Coronavirus:* Children with HCoV had the lowest mean age (5.1 months) of all virus groups. The mean DSS was 8.6, which was significantly lower than for RSV or HBoV. The admission rate was 10/24 (41.7%), the lowest of all virus groups.
- *Influenzavirus:* The mean age at onset of disease for FLU was 15.1 months, which was significantly higher than for RSV and some other viruses. The mean DSS was 7.9, lowest of all virus groups and significantly lower than for RSV. None of the patients was treated with antiviral drugs like oseltamivir.
- *Adenovirus:* For HAdV, there were no significant differences compared to the other viruses with regard to DSS and admission rate.
- *Bocavirus:* The mean age of children with HBoV was 18.6 months, which was significantly higher than for RSV, RV and HCoV. The mean DSS was also highest (19.1), which was significantly higher than for FLU. The admission rate was 9/10 (90.0%). Although the admission rate was the highest of all virus groups, this difference was not significant. The mean number of days of nebulization therapy with salbutamol and ipratropium bromide was 2.2 (SD 2.2) days, highest of all, although this was not significant compared to the other viruses.
- *Parainfluenzavirus:* The mean DSS for children with PIV was 13.9, third highest after HBoV and RSV. The number of times that antibiotics were initiated was highest for PIV (6/9, 66.7%).

### Clinical symptoms and management of multiple viral infections

Patients with a confirmed viral ARI had a significantly higher DSS, fever, LOS, extra oxygen supply and antibiotic treatment than patients with a negative RT-PCR result (Table 6). Nebulizer therapy and the admission rate did not significantly differ between these groups.

**Table 6:** Clinical symptoms and management in RT-PCR negative, positive, single- and multiple ARI

	RT-PCR negative	RT-PCR positive	*p*-value^a^	Single ARI	Multiple ARI	*p*-value
DSS, mean	8.83	13.83	**0.000**	13.57	14.51	0.243
Fever n/total (%)	37/103 (35.9)	243/457 (53.2)	**0.002**	173/331 (52.3)	70/126 (55.6)	0.529
Admission n/total, (%)	74/103 (71.8)	342/457 (74.8)	0.530	252/331 (76.1)	90/126 (71.4)	0.300
LOS (days)	2.99	3.95	**0.003**	4.02	3.76	0.432
Oxygen supply (days)	0.85	1.98	**0.000**	1.99	1.96	0.912
Nebulizer therapy (days)	1.03	1.19	0.227	1.10	1.46	0.140
Antibiotics (days)	1.56	2.32	**0.041**	2.19	2.67	0.190

*ARI* acute respiratory tract infection, *RT*-*PCR* reverse-transcriptase real-time polymerase chain reaction, *DSS* disease severity score, *LOS* length of hospital stay

^a^ Significant differences are noted as bold

Within the group of viral confirmed ARI, children with single- and multiple viral infections did not significantly differ with regard to DSS, fever, admission rate, LOS, extra oxygen supply, nebulizer therapy and duration of antibiotic treatment when initiated (Table 6). Sub analysis per group was performed for RT-PCR negative, single-, dual-, triple- and quadruple infections. No significant differences were found (data not shown).

A sub analysis of the five most common dual viral combinations (RSV/HCoV, RSV/RV, RSV/HAdV, RSV/hMPV, RSV/PIV) was performed in order to investigate whether these groups differed in clinical symptoms and management. There were no significant differences between the groups with regard to DSS (*p* = 0.958), admission rate (*p* = 0.318), LOS (*p* = 0.906), extra oxygen supply (*p* = 0.456), nebulizer therapy (*p* = 0.210) and antibiotic treatment (*p* = 0.339) (data not shown).

## Discussion

In this study, we investigated clinical presentation, management and outcome in a large cohort of patients with viral ARI and correlated these findings to the specific virus that was established by RT-PCR. Despite some significant differences, no clinically recognizable pattern per virus group was found. In addition, we showed that children with single- and multiple viral ARI did not differ with regard to clinical outcome.

### Single infections

The high number of RSV positive children, their young age, high admission rate and high DSS was expected since RSV is well known to have a great disease burden in young children [20]. RV usually is the most frequently found virus in young children and Enteroviridae peak in late summer and autumn [21]. However, in our study, RV was not frequently found as a single pathogen, possibly due to the sampling period in the winter. The high admission rate and moderate DSS stresses the growing evidence that RV is associated with a more severe ARI in young children [22–24].

In our study, clinical data of patients with hMPV did not differ to patients with other viruses. This is in line with literature, in which patients with RSV and hMPV were virtually indistinguishable with regard to symptoms and laboratory findings [25]. We did not find the typical male to female ratio of two to one, as reported earlier [26].

For the Coronavirus group, DSS, percentage of hospitalizations, the number of days with extra oxygen and the number of days with nebulization was low, suggesting a mild disease course. This was in contrast with the relative high median number of days in hospital. Only one specific patient was responsible for this effect. It was a 2 year old boy with a double sided pneumonia, DSS 19, maximum CRP 51 mg/ml, treated with intravenous antibiotics for 7 days.

The mean age of children positive for FLU was relatively high and most children were infected during the second winter season in their life. A possible explanation for this phenomenon is that the influenza-season lasts only a few weeks during a winter season [21]. As adults are also frequently infected, young children may be protected by circulating maternal antibodies against FLU during the first months of their life [27]. The low DSS for FLU was also remarkably as FLU is considered a potential virulent pathogen, especially in young children [2]. Possibly our inclusion criteria (children with ARI) may miss children with fever without a source or a sepsis like syndrome as is frequently seen in young children with influenza. Another important note is that our inclusion period was before the FLU-A H1N1 2009 pandemic occurred. The circulating FLU-A strains have changed in composition and this may have an effect on the clinical presentation of FLU nowadays. A recent study showed a more severe disease course in children with FLU-A compared to FLU-B [28].

The DSS for HBoV was high in our study. Similar results were found in a recent study showing that HBoV as a single pathogen can cause severe ARI [29]. The mean age of children with HBoV in our study was significantly higher than for children with RSV, RV and HCoV, which has not been reported before. A possible explanation is again protection by maternal antibodies. As reviewed by Jartti, protection by vertical antibody transfer is common at age < 2 months. After this age HBoV antibody-titers decline and are lowest at age 6–12 months. After 12 months seroprevalence of HBoV increases again until age 6 years. At that time almost all children have circulating HBoV antibodies [30].

Apneas are an important concern in young children with bronchiolitis. In our study, apneas occurred in seven out of 200 (3.5%) RSV single infections, comparable with data found in a recent review [31]. However, apneas occurred also in non RSV-infections (4/131, 3.1%). The clinical data and risk factors for children with apneas have been published elsewhere [18].

Although we showed some significant differences in clinical data between the virus groups, a specific clinically recognizable pattern per virus group could not be defined. All virus groups showed overlapping clinical symptoms.

### Multiple infections

Patients with a positive RT-PCR result were different from children with a negative RT-PCR result, except for admission rate and nebulization therapy (Table 6). A possible explanation is that asthma patients were not excluded in this study and nebulization therapy is sometimes started as test treatment in children with ARI and wheezing episodes. Patients with multiple infections were significantly older than patients with single infections, as is also previously reported [11, 32]. A possible explanation is a higher daycare attendance in older children, where crowding of children leads to virus transmission [33, 34]. Indeed, in our study daycare attendance appeared more often in children with a multiple infection.

### General discussion

Patients could be included multiple times in our study. To ensure that this was not in the same period of illness, an interval of at least 14 days between two NWS samples was chosen. In a sub analysis of the repeat cases, RT-PCR showed different viruses in 34 out of 35 patients between the first and second illness period. In one patient, both NWS were positive for RSV-A, but these samples were taken in different years. In 22 out of these 35 patients, RT-PCR was positive for multiple viruses.

There is increasing interest in the importance of viral load. Whether viral load, determined by cycle threshold values of RT-PCR assays may contribute to disease severity and/or to a better understanding of the role of multiple infections lay outside the scope of this study. This subject will be addressed in a separate paper.

A limitation of this study is the small number of patients in some virus groups, even after clustering of viral subtypes. This might have led to over- or underestimation of some effects. The clustering of different virus subtypes itself could potentially lead to underestimation of some more harmful subtypes. Some investigators showed a more severe disease course of RV subtype C [35], while others found a similar disease severity between subtypes A and C [36]. Our RT-PCR assay could not differentiate between different subtypes of RV. For RSV, an equal disease severity between the subtypes A and B is assumed [37]. We clustered FLU-A and FLU-B, and as mentioned above, inclusion of patients was before the FLU-A H1N1 2009 pandemic occurred. Secondly, bias may have been introduced in our study since most children were referred to the hospital only after initial assessment by a primary care physician, as is common in the Dutch healthcare system. Therefore, patients with milder disease may be underrepresented; this is also reflected in the high admission rate of 76.1% in single-infections and 74.3% in all ARI’s in this study. We used a modified scoring system to avoid subjective terms like moderate or severe. A concern in the interpretation of clinical severity using a DSS is the lack of uniformity between scoring systems for young children with ARI in literature. The severity score of Gern et al. was also used in a study correlating viral load and disease severity of RSV patients [38]. We also used a modification of this scoring system in a recent study [18]. Another concern is the lack of uniformity of case-definitions. A strict definition of URTI (ear, nose, throat region) or LRTI (bronchi and lung tissue) is difficult in young children, since classical criteria like tachypnea and hypoxia are not restricted to LRTI.

## Conclusion

In conclusion, clinical management and outcome in children with ARI are not determined by the type of virus. Children with one specific virus do not have a specific clinically recognizable pattern and children with single- and multiple viral ARI are clinically indistinguishable. LOS is determined by duration of extra oxygen supply or need for nebulizer therapy. The impact of RT-PCR for special indications is outside the scope of this paper as is the role of RT-PCR for other clinical purposes such as management of cohorting of inhospital patients. However, at this moment, for the general pediatric patient management the impact seems limited. In these settings, RT-PCR assays should be restricted to pathogens for which therapy is available, e.g. the clinical course can be influenced, such as for RSV, FLU and *Bordetella pertussis*.

## Additional file

Additional file 1: Table S1. Modified disease severity score after Gern [17, 18]. (DOCX 14 kb)

## Abbreviations

ARI: Acute respiratory tract infection
DSS: Disease severity score
FLU: Influenzavirus
HAdV: Human Adenovirus
HBoV: Human Bocavirus
HCoV: Human Coronavirus
HMPV: Human Metapneumovirus
LOS: Length of hospital stay
PIV: Parainfluenza virus
RSV: Respiratory syncytial virus
RT-PCR: Real-time reverse transcription-polymerase chain reaction test
RV: Rhinovirus
URTI: Upper respiratory tract infection
