# Supplementary material for: Single- and multiple viral respiratory infections in children: disease and management cannot be related to a specific pathogen
Source: BMC Infect Dis. 2017 Jan 11;17:62. doi: 10.1186/s12879-016-2118-6 (PMC5225597; doi:10.1186/s12879-016-2118-6)
Supplement: Additional file 1: Table S1. — Modified disease severity score after Gern [17, 18]. (DOCX 14 kb) [file 12879_2016_2118_MOESM1_ESM.docx]

**Additional file 1: Table S1. Modified disease severity score after Gern^17, 18^**

Symptom point score^a^

Fever 1

Cough 1

Rhinorrhea 1

Duration of illness > 4 days 1

Apnea 3

Wheezing 5

Hypoxia 5

Retractions 5

Tachypnea 5

^a^ maximum score is 27
